# A Preliminary Evaluation of Virginia Fresh Match: Impacts and Demographic Considerations for Future Fruit and Vegetable Incentive Programs

**DOI:** 10.3390/ijerph19074367

**Published:** 2022-04-05

**Authors:** Sarah A. Misyak, Molly K. Parker, Meredith Ledlie Johnson, Sam Hedges, Elizabeth Borst, Maureen McNamara Best, Valisa E. Hedrick

**Affiliations:** 1 Virginia Cooperative Extension, Department of Human Nutrition, Foods and Exercise, Virginia Tech, Blacksburg, VA 24061, USA; meredil@vt.edu; 2 Department of Human Nutrition, Foods and Exercise, Virginia Tech, Blacksburg, VA 24061, USA; pmolly95@vt.edu (M.K.P.); vhedrick@vt.edu (V.E.H.); 3 Local Environmental Agriculture Project, Roanoke, VA 24015, USA; hedgesjs@leapforlocalfood.org (S.H.); maureen@leapforlocalfood.org (M.M.B.); 4 Virginia Community Food Connections, Fredericksburg, VA 22404, USA; elizabethborst@gmail.com

**Keywords:** fruits and vegetables, nutrition incentives, Supplemental Nutrition Assistance Program, program evaluation

## Abstract

The purpose of this communication is to describe the preliminary evaluation of the Virginia Fresh Match (VFM) financial incentive program for fresh fruits and vegetables for Virginia Supplemental Nutrition Assistance Program shoppers and to determine if there were differences in incentive outcomes by race. In this cross-sectional study, a questionnaire was administered to shoppers using Virginia Fresh Match incentives at participating farmers markets and community-based food retail outlets. Repeated measures ANOVAs were used to detect differences in fruit and vegetable consumption between demographic groups over time. Chi-square tests were used to determine if there were associations between race and perceived impact of VFM incentives on making food last and the attribution of VFM incentives to changes in fruit and vegetable consumption frequency. Frequency of fruit and vegetable intake was significantly higher during VFM incentive use, with a difference of 1.17 ± 0.07 and 1.07 ± 0.07 on a Likert scale measure, respectively (*p* ≤ 0.001). There were racial differences in assertions that VFM incentives helped food to last. VFM incentives were effective at increasing fruit and vegetable consumption, but racial differences should be considered in the administration of VFM to avoid reinforcing systems or approaches that may contribute to disparities in food access and food security.

## 1. Introduction

Most Americans do not consume the recommended servings of fruits and vegetables [1,2]. Fruit and vegetable consumption and overall diet quality are lower for adults experiencing food insecurity compared to those experiencing food security [3,4]. This is also true for households participating in the Supplemental Nutrition Assistance Program (SNAP) [5], the largest government nutrition assistance program in the United States. 

The COVID-19 pandemic has strained supply chains [6,7], resulted in increased levels of food insecurity that disproportionately impacted households with children [8,9], and highlighted racial disparities in food access [10]. SNAP use, in general, appeared to be somewhat protective against food insecurity during the COVID-19 pandemic [11,12]. However, during the same time, those experiencing food insecurity were more likely to reduce their consumption of fruits and vegetables [13].

Shopping at farmers markets has been positively associated with fruit and vegetable intake [14], but cost is a barrier for those experiencing food insecurity [15]. Virginia Fresh Match (VFM) is a Food Insecurity Nutrition Incentive (FINI) Program—a funded, statewide network of farmers markets and food retail outlets that provides nutrition incentives for fruits and vegetables at the point of purchase to SNAP customers. VFM was established by two Virginia non-profit organizations, Virginia Community Food Connections and Local Environmental Agriculture Projects [16], after receiving a USD 1.8 million FINI grant in 2018. As of 2021, this network was made up of approximately 85 food retail outlets, including farmers markets and community-based food retail stores [16]. FINI-funded initiatives, such as the Double Up Food Bucks program out of Michigan and SuperSNAP out of North Carolina, have been shown to increase SNAP shopper expenditures on fruits and/or vegetables at farmers markets and grocery stores [17,18,19]. By providing nutrition incentives, VFM was uniquely suited to help address concerns with food access for SNAP shoppers living in the state of Virginia during the COVID-19 pandemic.

Through VFM, SNAP benefits spent by shoppers to purchase fresh fruits and vegetables are matched dollar for dollar. During the COVID-19 pandemic, VFM incentives were intended to increase the affordability of fresh fruits and vegetables and to provide important food access points for SNAP shoppers [20], as other incentive networks experienced increases in the number of customers seeking to use nutrition assistance benefits [21]. Given awareness of the disproportionate impact of the COVID-19 pandemic on the food security status of households with children as well as Black and Hispanic households [22], an evaluation of the impact of VFM incentives through a racial and household lens was warranted.

The purpose of this evaluation was to determine: (1) if VFM incentive use increased the frequency of fruit and vegetable consumption by SNAP and Pandemic-Electronic Benefit Transfer (P-EBT) shoppers in Virginia and (2) whether there were differences in changes in frequency of fruit and vegetable consumption among VFM incentive users given noted health disparities in food insecurity by race, gender, and household status before and during the COVID-19 pandemic. This evaluation can help drive VFM administrative and outreach strategies to vulnerable and/or minoritized groups.

## 2. Materials and Methods

This study utilized a cross-sectional design to reach a sample of the approximately 34,000 VFM incentive users at 85 retail outlets during 2021. A one-time questionnaire, available in English and Spanish, was administered to a purposive sample of VFM incentive users at 25 Virginia farmers markets and retail food cooperatives between August and October of 2021. The 16-item questionnaire was available in paper and online formats and took approximately 10–15 minutes to complete. Participants were provided with information on the questionnaire that the evaluation posed no more than minimal risk, that participation was voluntary, participants’ identities would be kept anonymous, and that consent was implied based on completion of the questionnaire. Farmers market staff were available to assist with completion of the paper questionnaires. Online questionnaires were completed by participants without assistance. Questionnaire participants were entered into a raffle for the possibility of winning 1 of 10, USD 100 gift cards.

In order to participate in VFM, shoppers had to use SNAP benefits at a participating retail outlet. To be eligible for the study, participants were asked to confirm they had used SNAP and/or P-EBT benefits (temporary nutrition assistance benefits on an electronic benefits transfer card provided by the United States Department of Agriculture (USDA) to compensate for the loss of school meals for students eligible for free or reduced meals with school closures during the pandemic) [23] within the last 30 days. The questionnaire was developed by program evaluators and revised based on community partner feedback. The questionnaire was deliberately short to meet the needs of farmers market managers and staff and to be more accessible to participants with limited literacy. 

Fruit and vegetable consumption were each measured with two questions on the same questionnaire asking for the frequency of consumption before using VFM incentives and while using VFM incentives. Likert scale answer choices ranged from 0 to 5, and respectively included I rarely ate fruit/vegetables, less than 1 time per day (a couple of times a week), 1 time per day, 2 times per day, 3 times per day, or 4 or more times per day. Participants were asked whether changes in fruit and/or vegetable consumption were due to VFM incentives, with answer choices including yes, partially, and no. To assess a potential impact of VFM incentives on food security status, participants were asked whether VFM incentives helped their food to last when they did not have money to buy more. Answer options were often true, sometimes true, never true. Demographics included gender, household status (i.e., whether children were living in the household), racial identity, ethnicity, and educational attainment. 

### Data Analysis 

Descriptive statistics were used to describe characteristics of questionnaire respondents. Normality was assessed using the Shapiro–Wilk test. After normality was confirmed, differences in the frequency of fruit and vegetable consumption pre-VFM incentive use were determined using a one-way ANOVA. Differences between fruit and vegetable consumption frequency before using and during using VFM incentives were analyzed using paired t-tests for the entire sample. Repeated measures ANOVA with a Tukey post-hoc test was used to detect differences in fruit and vegetable consumption between demographic groups over time. Chi-square tests with a post hoc analysis of adjusted cell residuals for significant relationships were used to determine whether there were associations between race and perceived impact of VFM incentives on making food last when they did not have money to buy more and the attribution of VFM incentives to changes in fruit and vegetable consumption frequency. Analyses were conducted on participants with available data; sample sizes may vary due to missing data. Significance was set a priori at *p* ≤ 0.05. Statistical Package for the Social Sciences (SPSS, version 27, IBM Corp., Armonk, NY, USA, 2020) was used for all data management and analyses. This evaluation received a not human subjects research determination from the Virginia Tech Institutional Review Board.

## 3. Results

A total of 251 participants completed the questionnaire. Descriptive statistics of the participants are presented in Table 1. Of the participants, half reported receiving VFM incentives when using P-EBT benefits, and the majority had children under the age of 18 living in their household. Most of the participants were white, non-Hispanic/Latino, and female. 

Self-reported frequency of fruit intake was significantly higher during VFM incentive use, with a difference of 1.17 ± 0.07 on the Likert scale measure (pre-VFM = 1.4 ± 1.2, during-VFM = 2.6 ± 1.1; *p* ≤ 0.001). Self-reported frequency of vegetable intake was also higher during VFM incentive use, with a significant difference of 1.07 ± 0.07 on the Likert scale measure (pre-VFM = 1.6 ± 1.1, during-VFM = 2.7 ± 1.0; *p* ≤ 0.001). Of the participants, 66.9% (*n* = 168) said their change in fruit and vegetable consumption was, or was partially (21.9%, *n* = 55), due to the use of VFM incentives. Further, 52.2% (*n* = 131) and 26.3% (*n* = 66) indicated it was often true or sometimes true, respectively, that VFM incentives helped their food to last when they did not have money to buy more.

When examining differences in the frequency of fruit and vegetable consumption by race, gender, and household status pre and during VFM incentive use, almost all groups demonstrated significant increases in frequency of fruit and vegetable consumption (*p* ≤ 0.001 for all except males, *p* = 0.003 and non-binary or other genders *p* = 0.208). No significant differences were found between groups for fruit or vegetable intake; however, white participants started at a significantly lower frequency of vegetable consumption compared to the other/multiple race group (*p* < 0.05), though not from Black participants. See Table 2.

There was a significant relationship between race and whether VFM helped food to last when there was not money to buy more, X^2^ (4, *n* = 223) = 11.4, *p* = 0.022. Based on Chi-square analysis with adjusted residuals, the proportion of white participants who indicated it was often true that VFM incentives helped food to last (*n* = 75, 48.4%) was significantly lower than expected, and the proportion of white participants indicating it was never true that VFM incentives helped food to last (*n* = 32, 20.6%) was significantly higher than expected. The proportion of participants from other or multiple racial groups who indicated it was often true that VFM incentives helped food to last (*n* = 18, 78.3%) was significantly higher than expected. There was no significant association between participants attributing changes in frequency of fruit and vegetable consumption to VFM incentives and race, X^2^ (4, *n* = 227) = 8.4, *p* = 0.077.

## 4. Discussion

This evaluation examined whether VFM incentive use increased the frequency of fruit and vegetable consumption by SNAP and P-EBT shoppers in Virginia and if there were differences in changes in consumption between groups. Shoppers using VFM incentives increased their frequency of fruit and vegetable consumption regardless of race, gender, or household status. However, a lower proportion of white participants than expected indicated that VFM incentives helped food to last when they did not have money to buy more. This indicates that less than half of white participants perceived VFM incentives as helpful for their general food security. In addition, white VFM incentive users started with the lowest fruit consumption frequency. Though white participants increased their consumption by similar levels as Black participants, the VFM incentive use was not enough to bring them to the same intake level as shoppers for the other/multiple races group.

Differences in perceptions of VFM incentives to help food last when there was no money to buy more by white participants may indicate that white shoppers with lower food security status saw VFM incentives at farmers markets and community-based retail stores as viable food access options for fresh fruits and vegetables, whereas shoppers from racial minority groups experiencing lower levels of food security did not. This is consistent with previous research showing disparities in farmers market access by race, with members of racial minority groups perceiving lower food access through farmers markets than white shoppers [21,24,25]. Given that previous research has shown that Black populations with lower incomes were more likely to experience food insecurity than white populations with lower incomes [26], and the role of structural racism in food insecurity [27,28], strategies to decrease disparities in food access are warranted. Of note, the only group to not significantly increase their frequency of fruit and vegetable consumption were those identifying as non-binary or other for their gender. This may be due to the limited sample size (*n* = 5 for fruit consumption and *n* = 6 for vegetable consumption).

Participants largely attributed positive changes in fruit and vegetable consumption to the use of VFM incentives, and the majority of participants indicated VFM incentives helped their food to last when they did not have money to buy more. A previous study of proposed solutions to improve healthy food access reported that food-insecure participants were likely to support initiatives to decrease the cost of healthy foods [15], a condition met by VFM incentives. Overall, this manuscript adds to the growing number of studies demonstrating a positive effect of point of purchase financial incentives on fruit and vegetable consumption [29,30,31], though it does conflict with a previous FINI evaluation that demonstrated no changes in fruit and vegetable consumption [32].

There were several limitations to this preliminary evaluation. The evaluation design was adapted from a comparison trial throughout the FINI grant to a cross-sectional study using a questionnaire at the end of the grant period because of constraints with social distancing measures due to the COVID-19 pandemic. The questionnaire was also limited in the number of items to comply with community partner preferences. Conducting the questionnaire at farmers markets where market managers volunteered to assist with distribution, may have resulted in sampling bias. There was also unequal representation from races/ethnicities, with the majority of respondents identifying as non-Hispanic/Latino and white or Caucasian and a limited sample of Black participants. Additionally, participants could skip questions, especially on the paper questionnaires, resulting in different sample sizes per question and an especially low response rate for the question on gender.

## 5. Conclusions

This initial investigation showed that VFM incentives increased Virginia SNAP and P-EBT shoppers’ frequency of fruit and vegetable consumption regardless of race, gender, or household status. However, there were racial differences in assertions that VFM incentives helped food to last when participants did not have money to buy more. These racial differences should be considered in the administration of VFM to avoid reinforcing systems or approaches that may contribute to disparities in food access and food security.

### Recommendations

The results of this evaluation can be used by the VFM incentive network, which has received a Gus Schumacher COVID-19 Relief and Response grant, part of the new suite of incentive program funding from USDA known as GusNIP, which replaces the FINI grant program by the federal government [33]. The VFM network should consider increasing outreach efforts to shoppers from minority populations experiencing food insecurity who may not see incentive use at farmers markets and retail stores as a viable option for stretching their food dollars. Given that the decision to shop at farmers markets by shoppers from minority groups is influenced by the perceived lack of racial/ethnic diversity at markets [34], outreach efforts should be made to minority shoppers to confirm that Virginia farmers markets can be a diverse and welcoming setting. The VFM incentive network can consider either increasing the accessibility of spaces among existing network partners or recruiting additional network partners that serve shoppers from different racial backgrounds. A qualitative investigation of the perceptions of VFM customers for strategies to increase the accessibility of markets could add to existing literature highlighting insufficient communication about an incentive program as a key barrier to program expansion [35].

The results are consistent with previous research showing that recruitment strategy must be considered in order to reach SNAP shoppers with higher levels of food insecurity and lower income [36]. Many customers learn about financial incentives on site, and awareness of incentives is higher for shoppers living near a retailer that accepts incentives [37]. The VFM network can consider improving SNAP shoppers’ utilization of VFM incentives by increasing its number of information sources, which has been significantly associated with receipt of incentives in a study by Vericker et al. [19].

These results could also inform policy at the state and national levels. For example, the Virginia Food Access Investment Fund (VFAIF), through which minority and historically low-income and low-access communities can receive grants to fund food retail projects to expand food access [38], shows that incentives provided at VFAIF outlets have the potential to increase fruit and vegetable consumption of Virginia SNAP shoppers and that these incentives can help food to last when shoppers do not have money to buy more. On the national level, an evidence base for the benefits of incentives is important given the federal investment in GusNIP [30]. There has been a call for shared measures for evaluation of financial incentives for fruits and vegetables to identify effective models for addressing food insecurity and increasing fruit and vegetable intake among populations with low incomes [39]. These preliminary data for VFM incentives demonstrate racial differences in incentive outcomes among VFM incentive users, and thus, highlight the need to consider the role of race to avoid reinforcing systems or approaches that may contribute to disparities in food access and food security. There has also been an identified need for technical assistance and the development of a community of practice by FINI grantees [40]. Including racial equity as a key topic for technical assistance may be beneficial.

## Figures and Tables

**Table 1:** Virginia Fresh Match incentive participant characteristics.

Participant Characteristics (*n* = Participants with Demographic Data)	*n*	%
**Benefit type with incentive use (*n* = 251)**		
SNAP	126	50.0
P-EBT	86	34.5
SNAP and P-EBT	39	15.5
**Household status (*n* = 242)**		
No children under 18 years old in household	57	24.0
One or more children under the age of 5	47	19.0
One or more children between ages 5 and 18	128	53.0
One or more children under the age of 18	10	4.0
**Race (*n* = 251)**		
White or Caucasian	163	65.0
Black or African American	46	18.0
Other or more than one race	24	10.0
**Ethnicity (*n* = 238)**		
Hispanic/Latino	11	5.0
Not Hispanic/Latino	227	95.0
**Gender (*n* = 116)**		
Female	93	80.0
Male	17	15.0
Non-binary or other	6	5.0

SNAP = Supplemental Nutrition Assistance Program Use, P-EBT = Pandemic-Electronic Benefit Transfer.

**Table 2:** Changes in frequency of fruit and vegetable consumption from before to during Virginia Fresh Match incentive use by race, gender, and household status ^1^.

Participant Characteristics (*n*)	Fruit Intake (*n*)	Fruit Intake Frequency Pre-VFM (Mean ± SD)	Fruit Intake Frequency During VFM (Mean ± SD)	Mean Difference ± SE (*p*-Value) ^2^	Significance Between Groups F value (*p*-Value) ^3^	Vegetable Intake (*n*)	Vegetable Intake Frequency Pre-VFM (Mean ± SD) ^4^	Vegetable Intake Frequency During VFM (Mean ± SD)	Mean Difference ± SE (*p*-value) ^2^	Significance Between Groups F Value (*p*-Value) ^3^
**Race**										
White	140	1.25 ± 1.13	2.40 ± 1.07	1.15 ± 0.09 (*p* < 0.001)	F = 1.605 (*p* = 0.203)	147	1.46 ± 1.08 ^a^	2.64 ± 0.95	1.18 ± 0.08 (*p* < 0.001)	F = 2.446 (*p* = 0.089)
Black	41	1.76 ± 1.37	2.90 ± 1.09	1.14 ± 0.18 (*p* < 0.001)		43	1.74 ± 1.22 ^ab^	2.53 ± 1.22	0.79 ± 0.20 (*p* < 0.001)	
Other	20	1.60 ± 1.35	3.20 ± 0.95	1.60 ± 0.23 (*p* < 0.001)		20	2.15 ± 1.18 ^b^	3.30 ± 0.86	1.15 ± 0.15 (*p* < 0.001)	
**Gender**										
Female	85	1.64 ± 1.16	2.80 ± 1.09	1.16 ± 0.12 (*p* < 0.001)	F = 0.665 (*p* = 0.516)	85	1.68 ± 1.10	2.75 ± 1.02	1.07 ± 0.13 (*p* < 0.001)	F = 0.185 (*p* = 0.831)
Male	15	1.67 ± 1.50	2.73 ± 1.10	1.07 ± 0.30 (*p* = 0.003)		16	1.81 ± 1.22	2.75 ± 1.13	0.94 ± 0.23 (*p* = 0.001)	
Non- binary or other	5	2.80 ± 1.30	3.40 ± 0.89	0.60 ± 0.40 (*p* = 0.208)		6	2.00 ± 1.90	2.83 ± 1.47	0.83 ± 0.48 (*p* = 0.141)	
**Household Status**										
No children	47	1.38 ± 1.15	2.32 ± 1.04	0.94 ± 0.14 (*p* < 0.001)	F = 1.515 (*p* = 0.222)	49	1.55 ± 0.96	2.39 ± 0.95	0.84 ± 0.12 (*p* < 0.001)	F = 1.788 (*p* = 0.170)
Children <5	50	1.58 ± 1.43	2.76 ± 1.20	1.18 ± 0.17 (*p* < 0.001)		51	1.63 ± 1.20	2.73 ± 1.10	1.10 ± 0.14 (*p* < 0.001)	
Children 5–18	113	1.35 ± 1.13	2.61 ± 1.05	1.26 ± 0.10± (*p* < 0.001)		119	1.61 ± 1.16	2.77 ± 1.00	1.17 ± 0.10 (*p* < 0.001)	

^1^ Likert scale answer choices for fruit/vegetable intake frequency ranged from 0 to 5, and respectively included I rarely ate fruit/vegetables, less than 1 time per day (a couple of times per week), 1 time per day, 2 times per day, 3 times per day, and 4 or more times per day. ^2^ Mean difference and standard error determined using paired t tests. ^3^ Difference between groups over time determined by repeated measures ANOVA. ^4^ Mean differences with varying letter superscripts represent significant differences between groups at baseline using ANOVA at *p* < 0.05 level. The values with different superscript letters (a and b) are significantly different. Values with ab are not significantly different from a or b.

## Data Availability

Data presented in this study are available on request from the corresponding author.
